# Years in Review: Recent Progress in Cellular Allergology

**DOI:** 10.1159/000444753

**Published:** 2016-03-09

**Authors:** Bernhard Kratzer, Winfried F. Pickl

**Affiliations:** Institute of Immunology, Center for Pathophysiology, Infectiology and Immunology, Medical University of Vienna, Vienna, Austria

**Keywords:** Allergy, T cells, Th17 cells, Regulatory T cells, Dendritic cells, Monocytes, Mast cells, Basophils, Eosinophils, Asthma

## Abstract

This review highlights the recent key advances in the biology of CD4^+^ effector T cells, antigen-presenting cells, Th17 and T regulatory cells, as well as immediate effector cells, such as mast cells, basophils and eosinophils, which are critically contributing to the better understanding of the pathophysiology of allergic diseases and are helping to improve their diagnosis and therapy. Some of the key advances with a direct impact on allergic asthma research and treatment are summarized.

## CD4^+^ T Helper Cells

CD4^+^ T cells are important to combat microbial pathogens, however, different types of pathogens require different responses. Therefore, different forms of CD4^+^ effector cells are required, which are characterized by the expression of specific surface molecules and the secretion of a specific set of distinct cytokines, and due to differential chemokine receptor expression have the capacity to enter into specific organs or organ systems [1]. These features are indispensable for the activation of other cell types, such as lymphocytes, macrophages and dendritic cells. Consequently, the role of CD4^+^ T cells is largely indirect, which gave them the name ‘helper’ cells [2]. Differentially polarized CD4^+^ T helper (Th) cells might also be involved in the decision of whether the immune response to an innocuous antigen, e.g. an allergen, either leads to sensitization or protection [3, 4].

In an attempt to impact on the effector functions and/or polarization of Th cells, Verheijden et al. [5] investigated the effects of galacto-oligosaccharides (GOS) on the production of the alarmin IL-33. IL-33 plays an important role in mucosal barrier tissues by impacting on Th2 cell function via the ST2 receptor [6]. GOS represent non-digestible oligosaccharides and are currently added to infant milk formulas with the aim of modifying the intestinal microbiome. Of note, dietary GOS application significantly reduced deoxynivalenol-induced IL-33 expression in the distal small intestine, which was paralleled by reduced ST2 expression on Th2 cells. Similarly, dietary supplementation with GOS reduced IL-33 and ST2 expression levels in the lungs and bronchoalveolar lavage fluids of house dust mite-allergic mice. Whether the biological effects of GOS supplementation result from alterations in the microbiome, the GOS-mediated protection of the intestinal barrier function – by maintaining the integrity of tight junctions – or direct interaction of GOS with epithelial and immune cells remains to be shown. Nevertheless, these data clearly show that the detrimental effects of disturbed barrier function can be counteracted by dietary GOS supplementation and that a reduction of IL-33 levels seems to be critically involved in this protective effect. A more direct approach was taken by Holvoet et al. [7] who cocultured human T cells, skewed towards the Th2 phenotype by a classic protocol in a 3-day culture with IL-4 and anti-CD40 mAbs [8], with a collection of 35 different probiotic bacterial strains and analyzed the secretion levels of signature cytokines. Of note, all strains downregulated IL-5 secretion levels similarly to or even stronger than LPS, which was used as a control. In contrast, IL-4 and anti-CD40 treatment rendered Th2-skewed cells more susceptible to IFN-γ production upon the addition of probiotics. The situation was different for IL-10 secretion. In fact, all probiotics induced IL-10 secretion, although to a much higher degree when cells were not prestimulated with IL-4 and anti-CD40 mAbs. The studies performed with cells obtained from healthy volunteers polarized towards the Th2 phenotype could be largely recapitulated with cells obtained from grass pollen-allergic individuals and ovalbumin (OVA)-specific murine splenocytes. Notably, the immunomodulatory effects of some probiotics stood out, such as that of *Lactococcus lactis,* which turned out to be a strong inducer of both IFN-γ and IL-10 secretion, and that of the *Escherichia coli* strains under investigation, which were identified as effective inducers of IFN-γ. The main finding was a certain degree of strain specificity in their immunomodulatory potency and differences in their potency to counterbalance established Th2 reaction profiles. Besides simply modulating cytokine profiles, it cannot be excluded that probiotics might also be involved in the reinforcement of the intestinal barrier, which would, however, require alternative tests for accurate evaluation. Nevertheless, the established in vitro assay allows the preselection of probiotics revealing a potentially beneficial cytokine pattern as candidates for detailed in vivo evaluations in the future [7].

The systemic and coordinated movement of lymphocytes in the body strongly depends on chemokine-based gradients induced by tissue- or cell-specific chemokine secretion and reciprocal chemokine receptor expression on the responding cell populations for efficient cell targeting [9]. Properly targeted lymphocytes are then prone to tissue-specific functions and also pathology, and these important immunoregulatory aspects have been studied by several groups. Barbarroja-Escudero et al. [10] analyzed peripheral blood T cells of patients with nonallergic asthma with respect to their chemokine receptor expression levels and related their findings to the dosage of fluticasone propionate (FP) medication applied. In high-dose FP-treated patients, CD3^+^ T cell numbers and percentages were found to be significantly reduced. Of note, untreated patients revealed a significant increase in both CCR6 and CXCR3 expression on CD8^+^CD25^+^ T cells, including CD25^high^ T cells, which seems to become normalized upon FP treatment, irrespective of the dosage applied. The chemokine receptors CCR2, CCR5 and CXCR4 did not show between-group differences. The fact that CCR6 is overexpressed in untreated asthma patients seems to be especially interesting, since CCR6 has been shown to target lymphocytes to the lungs, and might thus be a sign for poorly controlled asthma. The situation is more complex once patients are under medication since changes in receptor densities might be related to the systemic effects of FP treatment; however, they might also be related and mirror the severity of disease, since patients with severer forms of asthma are also more likely to receive standard- or even high-dose medication. Taking these confounders into account it was nevertheless interesting to observe increased CCR5 expression on several CD4^+^ and CD8^+^ T cell subsets derived from patients receiving standard-dose FP relative to those of healthy controls. Patients treated with high-dose FP revealed a significant reduction in CCR2 expression levels. CCR2^+^ T cells are known to be involved in the pathogenesis of severe asthma [11] indicating that either the effector memory pool becomes reduced or CCR2^+^ effector memory cells are confined to other compartments within the body. Thus, drug-induced changes in the chemokine receptor expression pattern might facilitate a better understanding of organ pathology and their amelioration in asthma [10].

Besides the respiratory tract, the skin and the eyes (conjunctiva) certainly belong to major target organs of hypersensitivity reactions [12]. It is therefore of special interest to better understand both the chemokine-receptor makeup and the effector cell potential of skin- and conjunctiva-homing lymphocytes. Skin-homing Th cells expressing the chemokine receptors CCR4, CCR6 and CCR10 preferentially produce IL-22 but not IL-17, which gave them the name Th22 cells [13, 14]. Such cells are enriched in the skin of atopic dermatitis (AD) patients. Since lesional skin in AD is known to be heavily colonized by *Staphylococcus* spp., Niebuhr et al. [15] were interested in whether staphylococcal enterotoxin B (SEB) and α-toxin would directly stimulate human Th cells for IL-22 production. In fact, SEB and α-toxin led to the enhanced secretion of IL-22 from Th22 cells, generated by coculture in IL-6- and TNF-α-containing medium, but also from memory T cells. Of note, T cells derived from AD or psoriasis patients or healthy controls secreted similar amounts of IL-22 when exposed to SEB or α-toxin. This indicates that skin-homing memory T cells might become hyper-stimulated by staphylococcal antigens once they have reached lesional skin colonized with large numbers of staphylococci, which in fact might critically contribute to the amplification of inflammation. In a related publication the function of skin-homing lymphocytes was studied by investigating individuals suffering from papular urticaria induced by flea bites. Of note, patients with papular urticaria presented with significantly more flea antigen-specific CLA4^+^ skin-homing T cells secreting IFN-γ, IL-4, IL-10 and IL-17 when compared to healthy controls. Interestingly, after more than 5 years of disease duration, skin-homing CLA4^+^ T cells lost their capacity to produce IL-4, while they largely maintained their IL-10 and IL-17 secretion capacity. Compatible with this hypothesis, the frequency of IFN-γ-positive T cells was reduced in both skin-homing and non-skin-homing lymphocytes in such patients [16].

Since chemokines and their receptors are not only responsible for lymphocyte trafficking but also contribute to the fine architecture of secondary lymphoid organs [17, 18], Shoji et al. [19] were interested in delineating the contribution of chemokines/chemokine receptors to the formation of inducible conjunctiva-associated lymphoid tissue (iCALT). The formation of iCALT was most efficiently induced by a combination of percutaneous and ophthalmic sensitization, revealing a significant accumulation of inflammatory cells including eosinophils and lymphocytes in sub-conjunctival tissues, which was accompanied by follicular lymphoid hyperplasia, including the accumulation of CD20^+^ B cells. Lymphoid follicles revealed an elevated expression of Th2 chemokines and corresponding chemokine receptors, including CCL17, CCL22 and CCR4, which were significantly higher in the keratoconjunctivitis than in the AD and healthy control group. PCR array-based evaluation of marker expression revealed marked overexpression of several chemokines and their respective receptors, including CCL20/CCR6, CCL17/CCR4 and CCL5/CCR3. The establishment of a reliable model for the induction of ophthalmic hypersensitivity along with the identification of associated chemokines and their receptors involved in that process not only contributes to the better definition of the pathology of such lesions, but also provides a collection of potentially helpful biomarkers for monitoring treatment-related changes in local pathology in this important target organ.

So far, allergen-specific immunotherapy (subcutaneous or sublingual) represents the only causal treatment of IgE-associated hypersensitivity reactions [20]. Biological model systems allowing the detailed investigation of hyposensibilization schedules including dose-response estimations and accurate monitoring of the respective immediate and long-term responses are therefore of utmost importance [21]. Along those lines van Rijt et al. [22] established a birch pollen-specific animal model by sequentially immunizing BALB/c mice with birch pollen extract, which led to airway hyperresponsiveness accompanied with hallmarks of allergic inflammation, such as bronchoalveolar lavage fluid eosinophilia and IL-5 hyperproduction, increased allergen-specific serum IgE levels as well as increased IL-4 and IFN-γ production by lung-draining lymph node cells upon restimulation. Subcutaneous immunotherapy led to a dose-dependent early decrease in Th2 cytokines, however, lung function was only ameliorated at later time points when allergen-specific IgG2a levels further increased. This study indicates that suppression of Th2 cytokines along with diminished eosinophil recruitment might not be sufficient to reduce airway hyperreactivity. Instead, amelioration of airway hyperreactivity might require a certain length of time in order to build up sufficiently high allergen-specific IgG2a titers. Of note, IgE levels increased until the sixth subcutaneous injection and then plateaued while IgG2a levels further increased, which led to gradually decreased IgE/IgG2a ratios from the seventh dose onwards. The authors have established a valuable model for studying the fine-tuning of allergen-specific immunotherapy for a highly human-relevant allergen.

## Th17 Cells

Th17 cells represent a newly identified Th cell subset [23, 24] characterized by the surface expression of CD161 [25] and the production of IL-17A, IL-17F, IL-21 and IL-22. Since IL-17 promotes GM-CSF production, its secretion also indirectly supports neutrophil generation [26]. Moreover, IL-17 promotes neutrophil recruitment to the site of inflammation by the induction of chemokine synthesis [26]. Neutrophils represent an important defense mechanism against extracellular microorganisms, such as bacteria and fungi, making Th17 cells an extremely important player during the defense of infections caused by pathogens such as *Staphylococcus aureus*, *Klebsiella pneumoniae* and *Candida albicans* [27, 28]. Besides this, Th17 cells play an important role in the pathogenesis of autoimmune and autoinflammatory diseases, including allergies [29, 30]. The study by Qin et al. [31] identified respiratory syncytial virus (RSV) infection of human bronchial epithelial cells as a major driver of leptin oversecretion, which in turn promoted Th17 and concomitantly suppressed Th2 subset differentiation, possibly induced by the regulation of Erk 1/2 phosphorylation. It has been shown previously that supernatants from RSV-infected human bronchial epithelial cells significantly increased both the differentiation of Th17 and Th2 subsets and decreased the differentiation of T regulatory (Treg) subsets. However, the molecules responsible for that process remained elusive. The authors have attained preliminary results from microarray screening experiments showing a significant elevation of leptin mRNA levels in RSV-infected human bronchial epithelial cells, which could be confirmed on the protein level in this publication. These are important findings since increased levels of Th17 subsets apart from Th2 subsets have been widely observed in asthma. Identification of leptin as a major driver of the escalation cycle leading to full-blown disease might open new and interesting avenues for potential therapeutic interference [32].

Along these lines, Naji et al. [33] compared circulating Th17 cell numbers and IL-17 levels produced by isolated peripheral blood mononuclear cells (PBMCs) of human atopic asthmatics before and after allergen inhalation challenge as well as atopic nonasthmatics and normal controls. They found a significant increase in circulating Th17 cells in asthmatics when compared to normal subjects. Moreover, PBMCs of atopic asthmatics produced significantly more IL-17 in vitro, mostly IL-17A, when compared to PBMCs derived from healthy controls. These results confirm earlier studies performed in murine models of allergen-induced airway disease and represent further proof of the importance of Th17 cells for the pathogenesis of allergic diseases and establish peripheral blood Th17 cell counts as an attractive monitoring tool for asthma. Very much in line with the previous study, Wang et al. [11] were interested in clarifying the molecular mechanisms guiding Th17 cells to the lungs in severe asthma. Therefore, the authors studied the effects of anti-CCL2 antibodies and CCR2 antagonist on the induction of allergic asthma, the levels of IL-17 and the frequencies of CCL2^+^ Th17 and Tc17 cells, as well as lung inflammation. Of note, blocking the CCL2/CCR2 axis significantly reduced recruitment of Th17 cells but not of Tc17 cells to the lungs, and also suppressed airway inflammation. This indicated that the CCL2/CCR2 axis orchestrates the recruitment of Th17 cells to the lungs, which, in turn, could be an attractive target for clinical exploration in the future.

While a number of disease states are caused or accompanied by an increase in Th17 cell numbers, recent evidence suggests that others, such as urticaria or food allergy, might in fact be inversely correlated with Th17 cell numbers. In support of this notion, Dhuban et al. [34] found significantly lower IL-17 production by Th17 T cells derived from the peripheral blood of food-allergic individuals when compared to PBMCs of healthy controls. For that purpose, PBMCs were cultured with peanut allergen Ara h 1 or 2, gliadin or tetanus toxoid for 9 days. Besides the reduction of IL-17-producing cells, a significantly lower percentage of CD4^+^FoxP3^+^ Treg was evident, suggestive of a concomitant reduction of the Treg compartment in children with food allergies. The authors propose that defects in IL-17 production and the resulting immune deviation might, in fact, predispose affected individuals to food allergies. Nevertheless, the critical contribution of defects in the generation and homeostasis of FoxP3^+^ Treg cells impacting on the sensitization to distinct foods cannot be excluded at present.

## Treg Cells

Different subsets of naturally occurring Treg cells have been defined and reviewed recently [35]. They express the coreceptor CD4, high levels of the high-affinity IL-2 receptor, CD25, and are positive for the transcription factor FoxP3 [36]. Treg cells are assumed to display a protective role in the immune response against allergens, with functional Treg deficiency being significantly associated with an increased risk for developing allergic diseases [37]. Tregs seem to play a crucial role during specific immunotherapy, especially in the early stages, where they apparently modulate the immune response by release of their suppressive cytokines TGF-β and IL-10 [38–40]. However, as it is now understood, the levels of CD4^+^CD25^+^FoxP3^+^CD127^–^ Treg cells already inversely correlate with allergen-specific serum IgE levels in allergic children [41]. In fact, Treg levels were especially low in patients with symptoms of AD and/or food allergy. The clear-cut results obtained by the authors might have to do with the fact that CD127 negativity besides FoxP3 positivity was used to unequivocally identify the Treg population, making the sometimes difficult task of discriminating between bona fide Treg cells and recently activated T effector cells easier and providing another example for a possible link between allergy and defective immunoregulation.

Since a complete lack but also incomplete deficiency of Tregs might predispose to allergic diseases [42], several studies evaluated protocols to potentially increase Treg numbers in vivo in the past [43]. Such strategies might be helpful for the development of a functional vaccine or be suitable as an adjuvant strategy for the treatment of allergic diseases [44]. Along these lines, Wu et al. [45] studied the effects of systemic treatment with 5-azacytidine in an OVA-based murine model of airway hyperreactivity. Of note, 5-azacytidine dose-dependently ameliorated airway hyperreactivity, as determined by enhanced pause measurement, decreased eosinophil infiltration of the lungs, lowered OVA-specific IgG1 and IgE serum levels, and decreased production of Th2 cytokines by splenocytes derived from drug-treated mice in a dose-dependent manner. A total of 9 doses of 5-azacytidine were applied twice weekly during OVA sensitization. This ensured drug interference with ongoing sensitization ultimately leading to the establishment of full-blown allergic disease. Further experiments depleting Tregs by the use of anti-CD25 antibodies completely reversed the beneficial phenotype, demonstrating the dependency of alleviation of airway inflammation from the induction of increased Treg numbers.

A different strategy to increase Treg numbers was explored by Yoshida and colleagues [46] by applying *Lacto-bacillus plantarum* to experimental animals via the oral route. It had been shown previously that the feeding of mice with *L. plantarum* is associated with higher Treg numbers in spleens and Payers patches and lower levels of serum IgE, however, the mechanism for this regulation has remained unclear so far. In their follow-up study, Tregs induced with the bacteria were adoptively transferred into mice sensitized to β-lactoglobulin. This maneuver decreased serum IgE levels and inhibited allergic skin reactions indicating that Treg cells not only influence systemic IgE levels, but might also interfere with mast cell degranulation [47]. Mechanistically, the biological effects induced by *L. plantarum* might be accomplished by Treg-based suppression of long-lived plasma cells [48] and/or OX40/OX40L-based inhibition of mast cell degranulation [49].

In contrast to the generally beneficial effects of Tregs during the physiological immune response against usually innocuous antigens such as allergens, the overabundance of such cells might, however, significantly interfere with diagnostic procedures aimed at identifying patients with (drug) hypersensitivity reactions. Consequently, positive results in in vitro lymphocyte transformation tests (LTT) might become masked by the presence of Treg cells deteriorating test sensitivity. Therefore, Srinoulprasert and Pichler [50] compared standard LTT with Treg-depleted LTT. Significantly, Treg depletion increased the LTT sensitivity to over 80% while maintaining test specificity. Of note, the Treg depletion methods applied, i.e. flow cytometric or immunomagnetic bead based, did not influence the beneficial outcomes.

## Monocytes and Dendritic Cells

Dendritic cells are the most important antigen-presenting cells, which capture the antigen in the periphery, traffic to their tissue-draining lymph nodes and process and present the captured antigens (allergens) to T lymphocytes through MHC-T cell receptor interactions [51]. Dendritic cells can respond to exogenous stimuli by upregulating costimulatory but also coinhibitory molecules (e.g. CD80, CD86 and OX40L) and cytokine secretion (e.g. IL-10 and IL-12), which critically divert the subsequent immune response into different directions [52]. In the initial years after their discovery, experimental procedures associated with the exploration of dendritic cell biology were quite laborious and cumbersome. The field of dendritic cell research was, however, revolutionized by the finding that highly purified peripheral blood monocytes can be differentiated towards dendritic-like cells [53, 54]. From that point on a number of studies focused on the function of antigen-presenting cells and their role during the polarization process in allergic diseases [55].

Along those lines, Ashjaei et al. [56] were interested in delineating the contribution of autologous dendritic cells to Th cell polarization. While coincubation of monocyte-derived dendritic cells (mdDC) obtained from polysensitized individuals with sensitizing allergens (Bet v 1 or Phl p 5) did not change their surface marker expression profiles, when compared to mdDC of healthy controls, such allergen-pulsed mdDC significantly drove Th2 cytokine production by autologous T cells. The authors found that this effect was in accordance with the sensitization profile of the patients under investigation, which was not observed when mdDC were pulsed with an unrelated allergen. Besides Bet v 1, the authors could show that Phl p 5 is also able to elicit Th2-type T cell responses in polysensitized individuals, while leaving the levels of IL-10 secretion unchanged. This either points to special features of the sensitizing allergens, which become imprinted onto the involved dendritic cells, as discussed and entertained by the authors, or rather mirrors already elevated allergen-specific T effector cell levels present in such specifically sensitized individuals. These findings, together with the observation of Dong et al. [57] that rapamycin-treated immature dendritic cells lead to the induction of Treg cells, add additional support to the concept that dendritic cells critically shape the T cellular immune response. Antigen-presenting cells receive important cues, such as ‘danger signals’, by specifically sensing their environment with their specific set of surface receptors, including histamine receptors [58]. The detailed study of the expression and function of histamine receptors on dendritic cells has the potential to shed important light onto the putative interplay between mast cell activation and the function of antigen-presenting cells [59]. Glatzer et al. [60] measured the expression of the different histamine receptors on monocytes and myeloid dendritic cells. They clearly demonstrated expression of histamine receptors 1, 2 and 4 (slightly higher on dendritic cells compared to monocytes) while histamine receptor 3 was consistently absent. Stimulation of myeloid dendritic cells with poly I:C in the presence of different doses of histamine led to the upregulation of CXCL1 and downregulation of TNF-α, CCL1, IL-6 and IP-10. In histamine-receptor blocking assays applying monoclonal antibodies, the authors could convincingly show that signaling via histamine receptor 2 and 4 on monocytes and myeloid dendritic cells is responsible for IP-10 downregulation. Reduced IP-10 expression might, in fact, promote Th2 polarization of T cells due to the absence of Th1 cytokines. In a similar effort, Lee et al. [61] were able to prove the functional importance of histamine receptor 1 in murine bone marrow-derived dendritic cells. During the maturation of dendritic cells, the presence of the histamine receptor 1 antagonists ketotifen and cyproheptadine led to a significant downregulation of TNF-α and IL-6 secretion, an effect not observed when the other two receptors (2 and 4) were blocked. However, histamine receptor 1 blockade did not alter the expression of the dendritic cell maturation marker molecules CD80, CD86, MHC class II, CD40 and CD11c, or the T cell stimulatory capacity of dendritic cells. The observed changes were attributable to alterations induced in the NF-κB signaling pathway involving c-Rel. In fact, with an adoptive transfer model of OVA airway hyperreactivity, the authors demonstrated that bone marrow-derived dendritic cells treated with ketotifen were able to prevent airway eosinophilia and Th2 cytokine expression when compared to vehicle-treated cells.

While dendritic cells are certainly the major antigen-presenting cells in our bodies, monocytes also carry out important antigen-presenting tasks and thereby either contribute to immunity or – in the case of hypersensitivity reactions – disease [62]. Monocyte subsets can be characterized by their differential CD14 and CD16 expression levels [63]. The ‘classical’ monocytes are strongly positive for CD14 and negative for CD16 (CD14^++^ CD16^–^ monocytes) and constitute the majority of all monocytes in healthy individuals. In contrast, monocytes positive for CD16 account for only 5–15% of all monocytes, but their frequency increases significantly in inflammatory conditions such as sepsis, tuberculosis and atherosclerosis. The upregulation of CD16 and the CD14 expression levels are used to discriminate between intermediate monocytes (CD14^++^CD16^+^) and so-called nonclassical monocytes (CD14^+^CD16^++^) [64]. Moniuszko et al. [65] investigated whether the phenotypes of different monocytes and CD4^+^ T cells in the peripheral blood of allergic patients suffering from house dust mite-sensitive allergic rhinitis differ from those of healthy controls, and whether changes in monocyte subsets and T cells would be interdependent. In contrast to the study of Ashjaei et al. [56], the authors found that in allergic individuals the phenotype of circulating monocytes is significantly different from that seen in healthy individuals. In fact, the upregulation of the monocyte mannose receptor CD206 and the IL-10R and concomitant downregulation of the IL-4R highlights the intensive cross-talk between innate and adaptive immune cells during allergen-specific immune responses in patients. Moreover, the CD16^+^ monocyte subsets, i.e. the CD14^++^CD16^+^ intermediate and the nonclassical CD14^+^CD16^++^ monocytes, were significantly enriched in patients, pointing to allergen-specific alterations within the compartment of antigen-presenting cells in allergic patients. These alterations were associated with significantly decreased frequencies of CD4^+^CD25^high^ T cells in allergic patients as compared to healthy controls. Since previous reports have already shown that the CD14^+^CD16^++^ and, to a lesser extent, the CD14^++^CD16^+^ monocyte subset play critical roles in asthmatic patients, protocols for their improved depletion were intensively sought for. In that context, it was shown that short-term glucocorticoid treatment efficiently depletes monocyte subsets. In an effort to reduce the potentially harmful glucocorticoid doses required, Grubczak et al. [66] combined glucocorticoid treatment with 1-α, 25-dihydroxyvitamin D_3_ treatment and observed synergistic effects with regard to monocyte subset depletion. Notably, in the presence of 1-α, 25-dihydroxyvitamin D_3_ much lower doses of glucocorticoids were required to achieve a similar degree of monocyte subset depletion as compared to glucocorticoid treatment alone, opening the door for attractive new treatment modalities.

## Mast Cells

Mast cells are typical sentinel cells within tissues, such as skin, the intestinal and airway mucosa, as well as the conjunctiva, which form immediate contacts with the external environment [67, 68]. Mast cells express the high-affinity IgE receptor, can become sensitized with allergen-specific IgE and, after crosslinking upon allergen encounter, release potent mediators, including histamine, leukotrienes, tryptase and others, and are responsible for the multitude of local and systemic reactions observed in IgE-associated hypersensitivity reactions [69]. Mast cell lines have been a valuable tool to study the biology of mast cells in the past. Since 2003 three major mast cell lines have been widely used for research purposes – HMC-1 (human mast cell leukemia-1) [70], LAD2 (laboratory of allergic diseases 2) [71] and LUVA (laboratory of University of Virginia) [72]. It turned out that the LAD2 cell line most closely resembles human primary mast cells, is genetically stable and has a constant doubling time if handled according to the original instructions. However, the degranulation capacity of LAD2 may deteriorate when cells are kept in culture for prolonged periods of time. Accordingly, the authors recommend switching to frozen stocks of early passages of LAD2 once a year [73]. One of the hallmarks of the detrimental tissue remodeling process in asthma is represented by a dramatic increase in the smooth muscle cell mass around the bronchial tree. In order to investigate whether or not smooth muscle cells become influenced by mast cells, the above-described HMC-1 cell line served as a model. Coculture experiments using conditioned medium of FcεR-α chain-transfected HMC-1 cells induced upregulation of IL-6 and IL-8 secretion in smooth muscle cells, which was even more pronounced if the mast cells were activated, e.g. by sensitization with IgE followed by stimulation with antigen. Furthermore, it was shown that the responsible factor(s) in conditioned medium of HMC-1 cells and acting on smooth muscle cells were most likely proteinaceous and delivered their activity by induction of the MAPK pathway [74]. These findings are paradigmatic for the important interactions between tissue-resident mast cells and their ‘contractile’ surroundings.

In order to reach and accumulate in target tissues, mast cells are endowed with important tissue migratory functions [75, 76]. An important facilitator in that respect is MMP-2 (matrix-metalloprotease 2). By studying RBL-2H3 and bone marrow-derived mast cells, Noma et al. [77] provided evidence that the NF-κB inhibitor (–)-DHMEQ reduces MMP-2 expression and, thus, decreases cellular invasion similar to the MMP inhibitor GM6001. This identified NF-κB as a potential target to reduce inflammation-induced mast cell invasion and accumulation.

Mast cell granules are also a rich source for proteases, among them also granzyme B, ready for release upon activation [67]. Recently, Rönnberg et al. [78] discovered that mast cells also express granzyme H, which has similar proteolytic activity as granzyme D. Granzyme H was initially described in T and NK cells, but its exact function in mast cells is elusive and remains to be shown.

Morphologically, mast cells are not always easy to quantify in clinical samples since special histologic treatment is required for their unequivocal identification. Therefore, Hagel et al. [79] resorted to quantifying tissue tryptase levels in endoscopically obtained colorectal samples of food-allergic individuals. Significantly, individuals with a manifest food allergy presented with clearly elevated tissue-tryptase levels when compared to healthy controls or food-allergic patients in remission. Thus, intestinal tissue tryptase levels in endoscopically removed samples might help to objectively confirm the diagnosis of a manifest food allergy and its remission. On a similar note, local expansion of mast cells, comparable to systemic mastocytosis, might be associated with an increased propensity for anaphylactic reactions. In that context, a very interesting case report described a patient who presented with a clonal mast cell activation syndrome upon ingestion of sulfites, which are frequently used as antioxidative food preservatives and are also present in many wines. No specific IgE elevation could be identified in this patient, thus it was speculated that mediator release from mast cells is the consequence of a hitherto unknown mechanism [80].

## Basophils

Besides mast cells, basophils are one of the key effector cells in allergic diseases since they express the high-affinity Fcε receptor and also play an important role during the Th2 skewing of the immune response [81, 82]. Historically, the discrimination between basophils and mast cells was challenging, however, in contrast to mast cells, basophils have a much shorter half-life (days instead of weeks), they circulate in peripheral blood but have also the capability to home to inflamed tissues [83]. The role of basophils during the development of Th2-driven allergic immune responses was reviewed recently [84], clearly demonstrating the importance of this cell type in allergic diseases but also as a potential target for specific therapy. In an attempt to better characterize basophils, Watson et al. [85] showed that human basophils also express the nicotinic acetylcholine receptors α_4_, α_7_, and also the α_1_, α_3_, α_5_ subunits on their surface. Moreover, they provided evidence that a synthetic nicotinic ligand, i.e. ASM-024, can inhibit basophil activation in vitro in a dose-dependent manner. Two patient groups with mild allergic asthma were treated with either 100 or 500 µM of ASM-024 on 9 consecutive days. Patients were challenged by exposure to nebulized specific allergen on day 8 and their peripheral blood basophils were assessed by determining CD203c expression levels 24 h later. Notably, basophils from patients in both treatment groups revealed significantly reduced basophil activation when compared to the basophils of placebo-treated patients, explaining previous findings that ASM-024 is able to reduce airway hypersensitivity. This study identifies nicotinic acetylcholine receptors as interesting targets to regulate allergen-specific basophil activation in vivo. In functional terms, a large number of studies have shown that quantification of basophil activation by flow cytometry in the basophil activation test (BAT) helps to assess IgE-driven immediate-type responses to allergens in allergic individuals [86]. Of note, both direct and indirect BAT represent highly sensitive laboratory tests to evaluate the sensitization of individuals to allergens or drugs [87, 88]. In the case of drugs, the drug itself or respective metabolites thereof might be the culprits leading to IgE-dependent basophil activation and degranulation. Fluoroquinolones represent an important class of antibiotics, inhibiting bacterial growth by interference with DNA gyrase, however, they frequently also cause allergic reactions. Mayorga et al. [89] could show that fluoroquinolones are highly photodegradable. This circumstance poses a considerable problem when BAT is performed at standard laboratory light conditions in order to identify fluoroquinolone sensitization. Thus, the authors strongly recommend performing BAT tests evaluating fluoroquinolone sensitization strictly protected from light, especially when moxifloxacin is the suspected culprit drug.

Although distinct laboratory conditions might negatively impact on the performance of the BAT, this test is clearly superior to simple determination of allergen-specific serum IgE levels. In contrast to double-blind, placebo-controlled food challenge, it represents a nonsubjective, nonbiased test system to evaluate treatment success in patients with oral allergy syndrome. Along these lines, Inuo et al. [90] could show that subcutaneous immunotherapy with Japanese cedar pollen extracts reduced the Japanese cedar pollen-specific and tomato fruit-specific BAT, however, this was only observed in patients who did not report tomato fruit-specific symptoms such as an oral allergy symptom. While the effect on tomato-induced BAT modulation is of great interest, the fact that the few patients with tomato oral allergy syndrome included into this study did not show decreased BAT might mirror high IgE sensitization levels not controlled by blocking antibodies and certainly warrants additional studies with larger patient collectives.

## Eosinophils

Eosinophilic granulocytes belong to the innate immune system and play an important role as effector cells in allergic inflammation [91, 92]. Activated eosinophils release cytotoxic molecules such as major basic protein, eosinophil peroxidase, eosinophilic cationic protein, lipid mediators and cytokines that are important in the defense against foreign pathogens but can also cause tissue damage and promote tissue remodeling [91, 92].

Especially in allergic asthma, it was shown that eosinophils are driving tissue remodeling, strongly contributing to one of the key pathologies of that disease [93]. Accordingly, Tang et al. [94] investigated IL-25 plasma levels and IL-25 receptor expression levels (IL17-RA and IL-17RB) on eosinophils derived from mild allergic asthma patients, atopic nonasthmatic patients and normal individuals. IL-25 becomes produced by epithelial cells and stimulates the production of IL-4, IL-5 and IL-13 by effector T cells, which, in the form of a positive feedback loop, leads to eosinophil recruitment. The authors could demonstrate that patients with allergic asthma presented with elevated IL-25 plasma levels and that the corresponding eosinophils revealed significantly higher expression levels of both IL-17A and IL-17B receptors (forming the IL-25 receptor) when compared to both atopic nonasthmatic individuals and healthy controls. These results show that IL-25 plays a pivotal role in allergic asthma, provides an explanation as to why persistent increases of eosinophils are observed in these patients, and might in fact represent one of the very initial factors driving asthma.

With regard to diagnostic procedures, the presence of eosinophils in the sputum is often taken as an indicator for the classification of the asthma phenotype involved and to show whether or not the disease is well controlled, i.e. is lacking frequent exacerbations [95]. Dente et al. [96] performed a 3-year longitudinal study in a group of 21 patients suffering from severe refractory asthma and queried the stability of sputum eosinophilia in that patient collective. Per patient, 10.1 ± 4.2 sputum samples were analyzed in the observation period. Of note, in these patients sputum eosinophilia seems to be a rather constant phenotype, with 87% of the patients presenting with elevated numbers of sputum eosinophils throughout the observation period, making this simple laboratory parameter a very reliable marker.

Allergic rhinitis has previously been shown to be responsive, among other therapeutics, to the intranasal application of steroids [97]. More recently, fluticasone furoate was shown to impact on both epithelial cell and on eosinophil survival. A novel mechanism by which steroids might impact on eosinophil survival has been characterized by Mullol et al. [98]. In fact, fluticasone furoate decreased the serum-induced production of GM-CSF, IL-6 and IL-8 by cultured nasal mucosa epithelial cells, which in turn led to reduced survival of enriched peripheral blood eosinophils in such conditioned media. These findings may give important new insights into the treatment options of inflammatory conditions involving the nasal epithelium in which eosinophilic infiltration is part of the problem. Thus, fluticasone furoate is not only antiinflammatory, but also decreases eosinophil survival.

## Summary and Conclusions

A number of exciting studies and models on cellular allergology were reported between 2013 and 2015 (table 1). Besides the meticulous identification and validation of new biomarkers for monitoring disease activity and the success of treatment in allergy, novel and highly interesting in vitro and in vivo model systems, for example to evaluate the activity of nutritional supplements or probiotics or the efficacy of specific immunotherapy, have been established. The reports published are excellent proof of the fact that both basic and applied research activities are cornerstones with which to improve our understanding of hypersensitivity reactions and their cure.

## Figures and Tables

**Table 1:** Studies related to allergic asthma and key findings

Cell type studied	Key findings	Important experimental details	Ref.
*Biomarkers*			
Circulating T cells	FP treatment induces a decreased expression of CCR2 on T cells as well as a reduction of total T cell numbers. Increased expression of CCR6 on CD3^+^ T cells in untreated asthmatics	40 nonallergic asthmatics and 16 sex- and age-matched healthy volunteers; 16 patients received 50–500 µg b.i.d. FP and 12 patients received >500 µg, 12 were untreated	10

Th17 cells	Increase in circulating Th17 cell percentages upon allergen inhalation challenge	16 atopic asthmatics, 10 nonatopic asthmatics and 10 healthy controls	33

Th17 cells/Th2 cells	Leptin is overexpressed in RSV-infected human bronchial epithelial cells. In turn, leptin induces Th17 but suppresses Th2 differentiation, showing how RSV may trigger asthma	Human bronchial epithelial cells overexpress leptin on the transcriptional and protein level. Leptin drives differentiation of PBMC towards Th17 cells in vitro	32

Eosinophils	Sputum eosinophilia was a stable phenotype in patients with noncontrollable (refractory) asthma during a 3-year observational period	Longitudinal study over 3 years including 21 adult asthmatics with 10.1±4.2 determinations per patient	96

Eosinophils	Increased IL-25 plasma levels and increased expression of IL-17RA and IL-17RB (IL-25R) in allergic asthmatics	14 atopic asthmatics, 15 atopic nonasthmatics, 14 healthy volunteers	94

*Treatment strategies and models*			
CD4 T cells	Dietary GOS reduce IL-33 secretion (lung and BAL) in a murine model of house dust mite-induced asthma	Balb/c mice intranasally sensitized to 1 µg of house dust mite allergen, challenged with 10 µg i.n. on day 7	5

Th17 cells	Blocking the CCL2/CCR2 axis in murine asthma reduces Th17 recruitment and suppresses airway inflammation	Balb/c mice sensitized with 100 µg of OVA and alum i.p. on days 0, 7 and 14 challenged on day 24 with 200 µg OVA i.t.	11

Treg cells	5-Azacytidine increases Treg cells in PB and diminishes airway hyperreactivity, pulmonary eosinophilia and OVA-specific IgE titers	Balb/c mice sensitized i.p. with 50 µg of OVA on days 1, 2, 3 and 14; aerosol challenge on days 14, 17, 20, 23 and 26. Injection of the drug twice weekly during sensitization	45

Monocytes	Vitamin D_3_ synergizes with glucocorticoids to induce apoptosis of CD14^+^CD16^++^ monocytes in asthmatics	Asthma patients and healthy volunteers	66

Basophils	Basophils express nicotinic receptors, and the nicotinic ligand ASM-024 decreases basophil activation in allergic asthmatics	Randomized, double-blind, placebo-controlled study. Receptor expression study included 9 subjects and 12 mild allergic asthmatics completed the intervention study	85

Th2 cells	Subcutaneous immunotherapy in birch pollen-allergic mice suppresses Th2-mediated eosinophilic airway inflammation early on. AHR becomes only reduced after induction of high titers of allergen-specific IgG2a	Balb/c mice, sensitized i.p. with birch pollen extract on days 0, 7 and 14 and treated for different periods with birch pollen extract as subcutaneous immunotherapy and aerosol challenge	22

*Potential new targets*			
Mast cells	Cocultured activated mast cells induce IL-6 and IL-8 secretion by human airway smooth muscle cells	In vitro study using the HMC-1 mast cell line and primary human airway smooth muscle	74
